# Identification and characterisation of mosquitoes from different locations in Qatar in 2017–2019

**DOI:** 10.1051/parasite/2021079

**Published:** 2021-12-16

**Authors:** Elmoubasher Abu Baker Abd Farag, Devendra Bansal, Khaled Mardini, Ali A. Sultan, Mohammed Hamad J. Al-Thani, Salih Ali Al-Marri, Mohammed Al-Hajri, Hamad Al-Romaihi, Francis Schaffner

**Affiliations:** 1 Ministry of Public Health Doha Qatar; 2 Friends of the Environment Center Doha Qatar; 3 Department of Microbiology and Immunology, Weill Cornell Medicine, Cornell University Doha Qatar; 4 Francis Schaffner Consultancy 4125 Riehen Switzerland; 5 National Centre for Vector Entomology, Institute of Parasitology, Vetsuisse and Medical Faculty, University of Zurich 8057 Zurich Switzerland

**Keywords:** Culicidae, Distribution, Vector species, Surveillance, Qatar, Arabic Peninsula

## Abstract

Mosquito-borne infections have considerable consequences for public health. The mere presence of a single case of vector-borne disease (VBD) introduces a risk to the local community particularly when associated with the compatible vector, host, and suitable environmental factors. Presently, there is no well-established vector control and surveillance programme in Qatar; therefore, the likelihood of VBDs spreading is undetermined. As a result, there is a pressing need to address this gap and enable successful management of VBDs. This study presents the results of three consecutive field surveys conducted between 2017 and 2019 with the aim of defining the types and distribution of mosquitoes that are of public health importance in Qatar. The results of the adult mosquito trappings show that the southern house mosquito *Culex quinquefasciatus* is the most widespread and abundant mosquito species, followed by *Cx. perexiguus*, both species representing a risk of West Nile virus transmission. All sampling methods show that the malaria vector *Anopheles stephensi* is widespread including in urbanised areas, suggesting a risk of local malaria transmission. The wetland mosquito *Aedes caspius* is also widespread, representing a risk of Rift Valley fever virus transmission. The dengue vector *Ae. aegypti* was not detected and can be considered neither widespread nor abundant, suggesting a minimal risk for local transmission of dengue, chikungunya and Zika viruses. Interestingly, the study detected *Culiseta longiareolata* for the first time in Qatar. Regular field studies are needed to further address the knowledge gaps in terms of distribution, ecology, and biting habits of different mosquito species currently present in Qatar to accurately assess the risk of mosquito-borne diseases.

## Abstract (fr)

Les maladies transmises par les moustiques posent de considérables risques en santé publique. La simple présence d’un cas de maladie à transmission vectorielle (MTV) introduit un risque pour la communauté locale lorsque associé à un vecteur, un hôte et des facteurs environnementaux compatibles. À ce jour il n’y a pas de programme de surveillance et de contrôle des vecteurs bien établi au Qatar, et de ce fait la probabilité de diffusion de MTV est indéterminée. C’est pourquoi il existe un besoin pressant de combler ce vide et de permettre une gestion effective des MTV. Ce travail présente les résultats de trois études de terrain successives conduites entre 2017 et 2019, dans l’objectif de caractériser les moustiques d’importance en santé publique et leur distribution au Qatar. Les résultats des piégeages d’adultes révèlent que le moustique domestique méridional *Culex quinquefasciatus* est l’espèce la plus répandue et abondante, suivie de *Cx. perexiguus*, les deux espèces présentant un risque pour la transmission du virus West Nile. L’ensemble des échantillonnages montrent que le vecteur du paludisme *Anopheles stephensi* est largement répandu y compris dans les zones urbanisées, ce qui suggère un risque de transmission locale du paludisme. Le moustique des zones humides *Aedes caspius* est également largement répandu, présentant un risque pour la transmission du virus de la fièvre de la Vallée du Rift. Le vecteur de la dengue *Ae. aegypti* n’a pas été détecté et peut être considéré ni répandu ni abondant, ce qui suggère un risque minimal de transmission locale des virus dengue, chikungunya et Zika. Il est intéressant de noter que cette étude a détecté la présence de *Culiseta longiareolata* pour la première fois au Qatar. Des études de terrain régulières sont nécessaires pour mieux combler les lacunes de connaissances en termes de distribution, écologie et comportement trophique des différentes espèces de moustiques présentes au Qatar, et d’évaluer plus précisément le risque de maladies transmises par les moustiques.

## الملخص

تشكل الأمراض المنقولة بالبعوض خطراً كبيراً على الصحة العامة، لاسيما في المناطق المعرضة لانتشار الأمراض السارية. في دولة قطر لا يوجد حالياً برنامج راسخ لمكافحة ومراقبة ناقلات الأمراض.ولذا هناك حاجة ماسة لسد هذه الفجوة وتمكين إدارة الأمراض المنقولة بالنواقل بنجاح. إن وجود، أو غياب، ناقل أو مسبب للمرض أو حالة مرضية محلية يظل يوحي بالخطر المحتمل للإصابة بالأمراض المنقولة بالنواقل..إن مجرد وجود حالة واحدة من الأمراض المنقولة بالنواقل يهدد المجتمع المحلي ولاسيما إذا ترافقت بوجود ناقل المرض المناسب أو العائل أو الظروف البيئية المناسبة. تستعرض هذه الدراسة نتائج ثلاثة مسوحات ميدانية متتالية أجريت بين عامي 2017 و 2019 بهدف تحديد أنواع وتوزيع البعوض ذي الأهمية الصحية العامة في قطر.قامت هذه الدراسة بتحليل وضع البعوض ذات الأهمية للصحة العامة في قطر خلال ثلاث دورات من الدراسات الاستقصائية الميدانية بين عامي 2017 و2019. وقد بينت النتائج المتحصلة من فخاخ البعوض البالغ أن بعوض المنزل الجنوبي أو البعوض خماسي الخطوط ( *Culex quinquefasciatus *) هو أكثر أنواع البعوض انتشاراً ووفرة، يليه بعوض ( *Cx. perexiguus *)، وكلا النوعين يمثلان خطراً على انتقال فيروس غرب النيل. وتظهر النتائج أيضاً أن ناقل الملاريا أنوفيليس ستيفنسي ( *Anopheles stephensi *) منتشر على نطاق واسع بما في ذلك في المناطق الحضرية، مما يشير إلى خطر انتقال الملاريا محلياً. البعوضة الرطبة إيديس كاسبيوس *Aedes caspius *)) كذلك تظهر أنها ذات انتشار على نطاق واسع، مما يمثل خطر انتقال فيروس حمى الوادي المتصدع. في هذه الدراسة، لم يتم الكشف عن الأيغوني ( *Ae. aegypti *) ويمكن بذلك اعتباره ليس وفيراً أو واسع الانتشار، ما يشير لضئالة احتمالية أو خطر للإصابة المحلية بفيروسات الضنك أو الشيكونغونيا أو زيكا. من المثير للاهتمام أن الدراسة رصدت بعوض كوليسيتا لونجياريلاتا *Culiseta longiareolata *)) لأول مرة في قطر. لابد من إجراء دراسات ميدانية منتظمة لمواصلة معالجة الفجوات المعرفية فيما يتعلق بتوزيع أماكن التكاثر البيئة ومناطق اللدغ المفضلة لدى مختلف أنواع البعوض الموجودة حالياً في قطر من أجل إجراء تقييم اكثر دقة لخطر الإصابة بالأمراض المنقولة عن طريق البعوض.

## Introduction

In recent years, the importance of vector-borne diseases (VBDs) has increased at the global and regional levels [29]. Several factors including the rapid growth of the human population, unprecedented urbanisation, increases in movement of humans and animals (travel and trade), and environmental challenges including climate change significantly impact the life cycle, the transmission and the geographical distribution of pathogens [17]. In non-endemic countries such as Qatar, the very first and crucial step in the prevention and control of VBDs requires the identification and appraisal of potential vector populations followed by mapping of the human and animal populations at-risk of acquiring (and transmitting) the pathogen. Currently, being a non-endemic country, the vector control and surveillance programmes were never well established in Qatar. Therefore, the Ministry of Public Health, Qatar, with technical assistance from Eastern Mediterranean Regional Office (EMRO) of the World Health Organization (WHO), have recently assessed the situation of vectors and their respective VBDs in Qatar [24, 25]. Analysis of the situation revealed a significant knowledge gap regarding the presence and distribution of mosquito species in different parts of the country, including rural-urban distribution. To address this issue, it was recommended to further strengthen Qatar’s technical capacity in the field of entomology, and in particular with emphasis on developing competencies toward vectors identification and surveillance. Subsequently, several field surveys were organised to assess the presence of key species of mosquitoes in different regions of Qatar, together with capacity-building activities.

At the time of the above-mentioned situation analysis, we conducted a literature review which included a total of nine studies, and together reported the occurrence of 20 mosquito taxa (Culicidae) in Qatar (Table 5) [25]. However, the majority of these 20 mosquito taxa were reported by a single publication. Moreover, in these cases, the authors often did not provide findings specifications, including for species described beyond their established distribution range, and thus their presence in Qatar requires further confirmation. Also, two studies reported taxa, i.e. *Culiseta* sp. and *Coquillettidia* sp., that remain yet to be identified at the species level. The literature review guided us in identifying the existing gap(s) regarding the distribution of different species of mosquito across various regions of Qatar. Furthermore, entomological reports from many neighbouring countries informed us about the presence of several mosquito species in the Middle East region (e.g. 49 species in Saudi Arabia [4]), which increases the probability of discovering other mosquito species (and sub-species) in Qatar. Therefore, we conducted field surveys to gather accurate and updated data about the presence and distribution of various mosquito species and carried out the risk assessment for mosquito-borne diseases in different regions of Qatar. Here, we report the main findings from the 3 sessions of field survey: (i) a longitudinal monitoring performed between August 2017 and August 2018; (ii) a series of samplings collected during the situation analysis mission, in September 2017; and (iii) a cross-sectional study undertaken in January 2019.

## Materials and methods

### Study area

**Figure 1:**
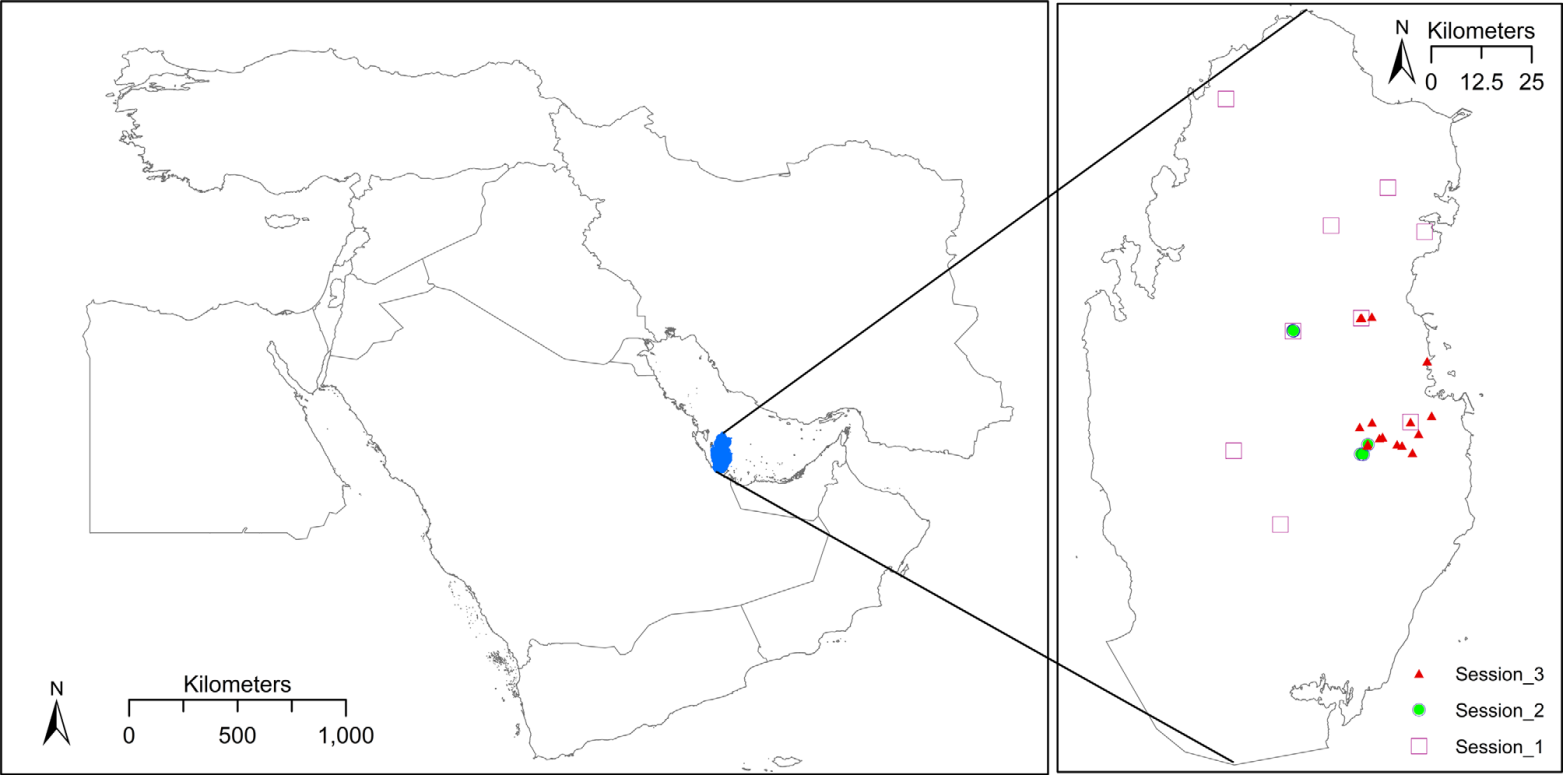
Study area and site locations. A. Location of the study area: Qatar, Middle East; B. Location of the study sites. White squares: Session 1 – longitudinal data, 2017–2018; Green circles: Session 2 – field survey, September 2017; Red triangles: Session 3 – cross-sectional field study, January 2019.

Qatar (24–26° N, 50–51° E) is a small peninsular country, located on the north-eastern coast of the Arabian Peninsula, Middle East (Fig. 1). The total area of Qatar is approximately 11,600 km^2^ and the total population is around 2,750,000 consisting of a large number of immigrants that varies from year to year (75.5% in the year 2015) [5]. Topographically, most of Qatar consists of a flat rocky plain (the highest point is 103 m), with a small range of limestone hills in the North–West and massive sand dunes in the South. The land is comprised of urban areas at 13%, rural areas at 84.5%, and has around 5.7% (670 sq. km in 2016) of agricultural land [5]. The country is divided into eight municipalities. Qatar’s climate is classified as a hot desert (Köppen-Geiger category BWh), with an annual mean temperature of 27.1 °C and mean rainfall of 72 mm (most rainfall is between October and May) [6].

### Field sampling

#### Session 1: Longitudinal sampling, 2017–2018

A series of repeated sampling (longitudinal) sessions were carried out to collect adult mosquito samples from across the country over a period of one year, to account for seasonal data. A total of nine locations were selected across the country to account for different environment sub-types that would influence mosquito breeding such as farms, gardening centres, and zoos (Table 1, Fig. 1).

**Table 1:** Location and characteristics of sampling sites [F1–F9: Longitudinal survey, session 1; Q01–Q20 Field surveys, session 2 (September 2017) and 3 (January 2019)], with sampling method, period, and number of samples analysed.

Site ID	Municipality	Location	Habitat	Latitude	Longitude	Method	Period	No. samples
F1	Al Doha	Widam Company	Garden centre	25.235981	51.485530	Adult trapping	Aug-17 to Aug-18	12
F2	Al Khor	Al Sidra Farm	Farm	25.675798	51.307606	Adult trapping		12
F3	Al Khor	Sewage Treatment Plant	Sewage basins	25.661767	51.517150	Adult trapping		9
F4	Al Khor	Umm Barkah	Farm	25.760504	51.434899	Adult trapping		13
F5	Al Rayyan	Al Rekkiya	Farm	25.006483	51.194028	Adult trapping		10
F6	Al Shahaniya	Al Dosari park and game	Zoo	25.439317	51.222233	Adult trapping		15
F7	Al Shahaniya	Umm Weshah	Farm	25.171333	51.089283	Adult trapping		8
F8	Al Shamal	Al Zobara	Farm	25.959183	51.072083	Adult trapping		9
F9	Umm Salal	Al Siletin	Farm	25.468589	51.375235	Adult trapping		11
Q01a	Al Shahaniyah	Al Dosari park and game	Basin beside fish pond, temporary	25.440457	51.222572	Larval sampling	Sep 17	1
						Larval sampling	Jan 19	1
Q01b	Al Shahaniyah	Al Dosari park and game	Covered cistern	25.440364	51.223533	Larval sampling	Jan 19	1
Q01c	Al Shahaniyah	Al Dosari park and game	Tyre (dry)	25.441110	51.222361	Larval sampling	Jan 19	1
Q02	Al Rayyan	Abu Nakhlah, sewage lake	Pond border with vegetation	25.164420	51.377165	Larval sampling	Sep 17	1
Q03	Al Rayyan	Abu Nakhlah, sewage lake	Marsh border	25.164499	51.373740	Resting catch	Sep 17	1
						Human landing catch	Sep 17	1
						Human landing catch	Jan 19	1
Q04	Al Rayyan	Abu Nakhlah, sewage lake	Isolated puddles outside embankment	25.163673	51.379603	Larval sampling	Sep 17	1
						Larval sampling	Jan 19	1
Q05	Al Rayyan	Abu Nakhlah, new village	Two metallic cisterns	25.185860	51.390198	Larval sampling	Sep 17	1
Q06	Al Doha	Nuaija	Container	25.249994	51.532889	Larval sampling	Jan 19	1
Q07	Al Rayyan	Abu Hamour	Flooded land with vegetation	25.209601	51.503795	Larval sampling	Jan 19	1
Q08	Al Doha	Al Waab	Park/garden	25.236129	51.485550	Adult trapping	Jan 19	3
Q09	Al Rayyan	Abu Sidra	Flooded land with vegetation	25.235270	51.399403	Larval sampling	Jan 19	1
Q10	Al Rayyan	Al Maqran	Wetland	25.224855	51.371550	Larval sampling	Jan 19	1
Q11	Al Rayyan	Abu Nakhlah, new village	Four containers	25.186946	51.389706	Larval sampling	Jan 19	1
Q12	Al Rayyan	Abu Nakhlah, new village	Flooded land with vegetation	25.183686	51.387945	Larval sampling	Jan 19	1
Q13	Al Doha	West Bay	Two road drains	25.372495	51.522716	Larval sampling	Jan 19	1
Q14a	Umm Salal	Al Silatin Agricultural Complex	Park/garden	25.469579	51.376009	Adult trapping	Jan 19	3
Q14b	Umm Salal	Al Silatin Agricultural Complex	Artificial rock pool	25.469011	51.373434	Larval sampling	Jan 19	1
Q15	Umm Salal	Umm Salal Ali	Five uncovered cisterns	25.471937	51.398886	Larval sampling	Jan 19	1
Q16	Al Rayyan	Industrial area	Road puddles with vegetation	25.199847	51.415835	Larval sampling	Jan 19	1
Q17a	Al Rayyan	Industrial area	One iron barrel	25.202058	51.422577	Larval sampling	Jan 19	1
Q17b	Al Rayyan	Industrial area	Worker house in construction	25.202093	51.422830	Resting catch	Jan 19	1
Q17c	Al Rayyan	Industrial area	Worker house, outdoor	25.202546	51.422650	Adult trapping	Jan 19	1
Q18	Al Rayyan	Industrial area, Labour camp	One basin/fountain and four road drains	25.167192	51.489907	Larval sampling	Jan 19	1
Q19	Al Rayyan	Industrial area	Worker house, outdoor	25.186026	51.455459	Adult trapping	Jan 19	1
Q20	Al Rayyan	Asian town	Wetland	25.183368	51.466513	Larval sampling	Jan 19	1

Adult mosquitoes were collected through MozzTech Mosquito Traps (Ridpest, Malaysia) baited with Octenol and CO_2_ that is produced by photocatalytic reaction of titanium dioxide exposed to black light. The traps were set for two consecutive nights each week between August 2017 and August 2018. The mosquitoes caught by this process were collected daily in the morning, and then frozen once transported to the laboratory for sorting and identification under a stereo microscope.

#### Session 2: Field survey, September 2017

To obtain an overview and insight about the mosquito breeding habits in Qatar, five sites previously known to local municipality’s pest control workers as common sites for mosquito breeding were inspected for three days (September 18–20, 2017) (Table 1, Fig. 1). Two strategies were used to collect larval samples: (i) using a net with a fine mesh and then transferring the samples to a 1-L white plastic tray for observation; and (ii) filling the tray by directly dipping it in water. Larvae and pupae collected using these techniques were transferred with water to a vial for transport to the laboratory. There, 4th instar larvae were transferred to a 70% ethanol solution and young larvae and pupae were kept until they grew to 4th instar or emergence of adults. In addition, resting catches were performed by using sweep nets around vegetation, and human landing catches were performed by netting around a person. In both cases, adults were collected from the net via a mouth aspirator and brought to the laboratory.

#### Session 3: Cross-sectional field study, January 2019

**Figure 2:**
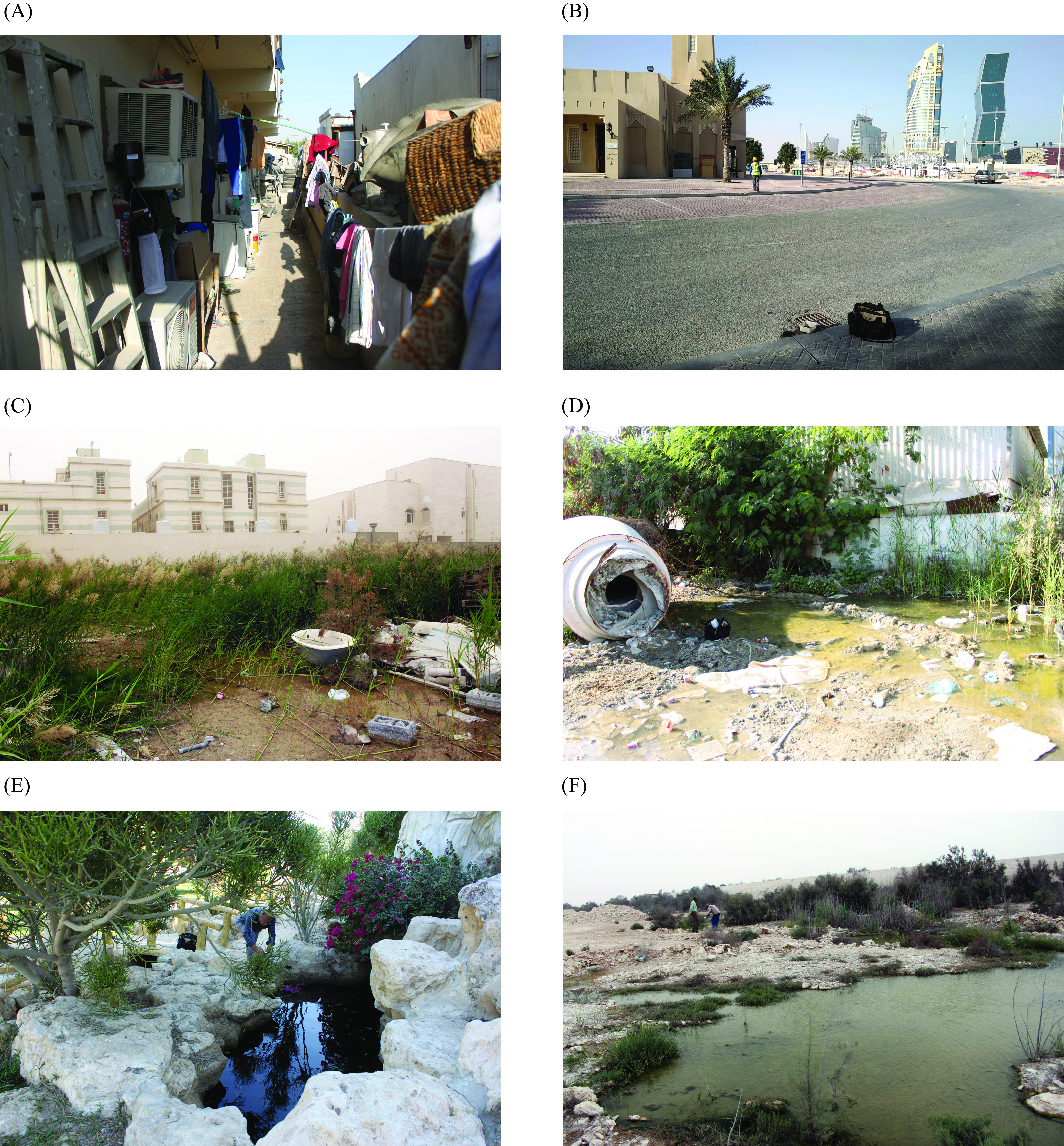
Examples of sites inspected for mosquitoes. A. Adult trapping at worker house, EVS trap (Q17c). Mosquito larval breeding sites: B. Road drain, breeding site for *Culex quinquefasciatus* (Q13); C. Flooded land in urban habitat, breeding site for *Anopheles stephensi*, *Culex perexiguus*, *Culex quinquefasciatus*, *Culex tritaeniorhynchus* (Q09); D. Flooded land in an industrial zone, breeding site for *Anopheles stephensi*, *Culex perexiguus*, *Culex quinquefasciatus* (Q16); E. Man-made container, positive for *Anopheles stephensi*, *Culex quinquefasciatus*, *Culiseta longiareolata* (Q14b); F. Wetland, breeding site for *Aedes caspius*, *Anopheles stephensi*, *Culex perexiguus*, *Culex pusillus*, *Culex quinquefasciatus* (Q04).

A cross-sectional study was conducted with the aim of updating the pre-existing database of the mosquito fauna of Qatar, for species presence at as many sites as possible. A total of 18 sites were selected across the country for collecting the mosquito samples. These sites were selected to ensure rapid collection and transport of the samples to the laboratory within a one-day trip. These sites covered all possible ranges of environments, e.g. urban building areas, farms, garden centres, industrial areas, sewage lakes, wetlands, worker houses, and zoos (Table 1, Figs. 1 and 2). All the samples were collected between January 15–23, 2019. The choices of sites were guided by municipalities’ pest control workers, satellite images and/or visually along roads in the course of journeys. Larval samplings, resting catches and human landing catches were performed at every selected site, as described for session 2. In addition, adult trapping was performed with CO_2_-baited traps (Fig. 2A), i.e. Heavy Duty EVS trap (BioQuip Products Inc., USA), CDC Mini Light Trap (BioQuip Products Inc., USA) and BG-Sentinel 2™ trap (Biogents, Germany). Traps were run overnight, and baited with dry ice at selected locations. Adults were collected with the trap net and brought to the laboratory, and frozen before identification.

### Mosquito identification

#### Morphology

Mosquito larvae and adults (females and males) were classified as belonging to a species or, if not possible, to a group of morphologically closely related species based on standard identification keys using stereomicroscope [3, 7, 11, 12, 23]. Several subsamples of mosquito larvae and adults were preserved in ethanol (larvae and immature exuviae, male genitalia) or pinned in an insect box (adults). Molecular identification by DNA isolation and amplification of the mitochondrial cytochrome oxidase subunit I gene (*COI*) for *Culex* sp. or of the ribosomal internal transcribed spacer 2 (*ITS2*) for *Anopheles* sp. was performed on only a small fraction of total specimens, as described elsewhere [16, 26]. New sequences were deposited in GenBank with accession numbers OL653979, OL654412, OL672837, OL672843, and OL672844. In addition, a rapid polymerase chain reaction (PCR) assay that uses polymorphisms in the second intron of the acetylcholinesterase-2 (*ACE2*) locus was run for the identification of specimens of the *Cx. pipiens* complex and possible hybrids [28].

## Results

### Longitudinal data, 2017–2018

Thousands of mosquitoes were collected in session 1, but the presence of considerable by-catches (attracted by the black light) and the poor quality of preservation did not allow all specimens to be properly sorted and identified. However, to obtain an estimate of sampling outcomes under our time constraints, we performed subsampling and analysed one randomly chosen sample per month and per site.

We analysed 99 samples, yielding detection of seven mosquito species or groups, the most abundant being *Culex quinquefasciatus* species group (*Cx. (Culex) pipiens* (Linnaeus, 1758), *Cx. (Cux.) quinquefasciatus* Say, 1823, and *Cx. (Cux.) perexiguus* Theobald, 1903, which are almost impossible to distinguish as dried – and often damaged – adults) detected at all sites, followed by *Anopheles (Cellia) stephensi* Liston, 1901 collected at four sites (Table 2). No other *Anopheles* species was detected here. *Culex quinquefasciatus* gr. was highly abundant almost all over the year, whereas *An. stephensi* showed medium abundance in Oct–Nov and Jun–Jul (Table 3). A third species, *Aedes (Ochlerotatus) caspius* (Pallas, 1771), was detected at three sites only and at several periods over the year, but in small numbers. In addition, the species *Culiseta (Allotheobaldia) longiareolata* (Macquart, 1838) was found at three sites.

**Table 2:** Relative abundance of mosquito species collected in the longitudinal adult monitoring, per site, August 2017–September 2018, according to one sample per month per site. One black dot = 1–10 individuals; Two black dots = 11–50 individuals; Three black dots = >50 individuals.

Site ID	*Aedes caspius*	*Anopheles stephensi*	*Cule quinquefasciatus* group	*Culex tritaeniorhynchus*	*Culex pusillus*	*Culiseta longiareolata*
F1	–	●	●●	–	–	–
F2	●	●●	●●●	–	●	–
F3	–	–	●	–	–	–
F4	●	●	●●	–	–	●
F5	–	–	●●●	–	–	–
F6	●	●	●	–	●	●
F7	–	–	●●	–	–	–
F8	–	–	●	–	–	–
F9	–	–	●	●	–	●

**Table 3:** Relative abundance and seasonality of mosquito species collected in the longitudinal adult monitoring, monthly, August 2017–September 2018, according to one sample per month per site. One black dot = 1–10 individuals; Two black dots = 11–50 individuals; Three black dots = >50 individuals.

Month & Year	*Aedes caspius*	*Anopheles stephensi*	*Culex quinquefasciatus* group	*Culex tritaeniorhynchus*	*Culex pusillus*	*Culiseta longiareolata*
Aug-17	–	–	–	–	–	–
Sep-17	–	–	●	–	●	–
Oct-17	–	●●	●●●	–	●	–
Nov-17	●	●●	●●●	●	–	–
Dec-17	–	●	●●●	–	–	–
Jan-18	●	–	●●●	–	–	–
Feb-18	–	–	●●●	–	–	●
Mar-18	●	●	●●●	–	–	–
Apr-18	●	●	●●●	–	–	●
May-18	●	–	●●●	–	–	–
Jun-18	–	●●	●●●	–	–	–
Jul-18	●	●●	●●●	–	●	–
Aug-18	–	–	●●	–	●	–

### Field studies, September 2017 and January 2019

In sessions 2 and 3, a total of 20 sites were surveyed with 6 samples collected in 2017, and 27 in 2019 (Tables 1 and 4). This comprises 20 larval samplings, 2 adult human landing catches, 3 adult resting catches, and 8 adult trappings. Larval samplings yielded 933 larvae and 97 pupae, and entrapped adult mosquitoes accounted for 20 males and 101 females.

**Table 4:** Mosquito species observed during our sessions 2 and 3 field surveys in Qatar, September 2017 and January 2019, per site. Within rounded parentheses: adults obtained by rearing of immatures; Within braces: number of traps. F = female; L = larva; M = male; P = pupa.

Site ID	Date	Method	Numbers and stages observed	Species
Q01a	20.01.2019	Larval sampling	3 L (1 F)	*Culex tritaeniorhynchus*
Q01b	18.09.2017	Larval sampling	20 L	*Anopheles stephensi*
			15 L	*Culex quinquefasciatus*
			25 L	*Culiseta longiareolata*
Q01b	20.01.2019	Larval sampling	3 L	*Culiseta longiareolata*
Q01c	20.01.2019	Resting catch	9 M, 4 F	*Culex quinquefasciatus*
Q02	20.09.2017	Larval sampling	40 L, 9 P (7 M, 10 F)	*Culex pusillus*
			6 L (1 F)	*Culex tritaeniorhynchus*
Q03	20.09.2017	Resting catch + Human landing catch	1 M, 2 F	*Aedes caspius*
Q03	17.01.2019	Human landing catch	1 F	*Aedes caspius*
Q04	20.09.2017	Larval sampling	56 L, 12 P (23 M, 19 F)	*Culex pusillus*
			2 L,1 P (1 M, 1 F)	*Aedes caspius*
Q04	17.01.2019	Larval sampling	1 L	*Aedes caspius*
			12 L, 5 P (4 M, 2 F)	*Anopheles stephensi*
			25 L, 10 P (6 M, 12 F)	*Culex perexiguus*
			50 L, 15 P (20 M, 18 F)	*Culex pusillus*
			1 L, 1 P (1 F)	*Culex quinquefasciatus*
Q05	20.09.2017	Larval sampling	6 L (1 F)	*Culex pusillus*
Q06	16.01.2019	Larval sampling	1 L (1 F)	*Culex perexiguus*
			15 L (3 M, 3 F)	*Culex quinquefasciatus*
Q07	16.01.2019	Larval sampling	22 L, 2 P (2 M)	*Culex pusillus*
Q08	17.01.2019	Adult trapping {3}	1 F	*Anopheles stephensi*
			1 M, 33 F	*Culex quinquefasciatus*
			9 F	*Culex tritaeniorhynchus*
Q09	17.01.2019	Larval sampling	1 L	*Anopheles stephensi*
			2 L (1 M)	*Culex perexiguus*
			48 L, 5 P (2 M, 5 F)	*Culex quinquefasciatus*
			4 L	*Culex tritaeniorhynchus*
Q10	17.01.2019	Larval sampling	25 L (1 F)	*Culex perexiguus*
			5 L (1 M, 2 F)	*Culex tritaeniorhynchus*
Q11	17.01.2019	Larval sampling	3 L (1 F)	*Aedes caspius*
			12 L	*Culex quinquefasciatus*
Q12	17.01.2019	Larval sampling	100 L, 5 P (3 M, 2 F)	*Culex perexiguus*
			5 L, 1 P (1 F)	*Culex tritaeniorhynchus*
Q13	19.01.2019	Larval sampling	180 L, 7 P (6 M, 1 F)	*Culex quinquefasciatus*
Q14a	21.01.2019	Adult trapping {3}	1 M, 17 F	*Culex quinquefasciatus*
Q14b	21.01.2019	Larval sampling	15 L, 2 P (2 F)	*Anopheles stephensi*
			32 L, 2 P (1 M, 1 F)	*Culex quinquefasciatus*
			25 L	*Culiseta longiareolata*
Q15	21.01.2019	Larval sampling	4 L	*Culex perexiguus*
			35 L	*Culex quinquefasciatus*
Q16	21.01.2019	Larval sampling	1 L	*Anopheles stephensi*
			1 L	*Culex perexiguus*
			18 L, 4 P (3 M, 1 F)	*Culex quinquefasciatus*
Q17a	21.01.2019	Larval sampling	4 L	*Culex quinquefasciatus*
Q17b	21.01.2019	Resting catch	4 M, 6 F	*Culex quinquefasciatus*
Q17c	22.01.2019	Adult trapping {1}	–	–
Q18	21.01.2019	Larval sampling	50 L, 14 P (8 M, 6 F)	*Culex quinquefasciatus*
Q19	22.01.2019	Adult trapping {1}	1 M	*Aedes caspius*
			2 M, 29 F	*Culex quinquefasciatus*
Q20	21.01.2019	Larval sampling	60 L, 2 P (2 F)	*Culex perexiguus*

**Figure 3:**
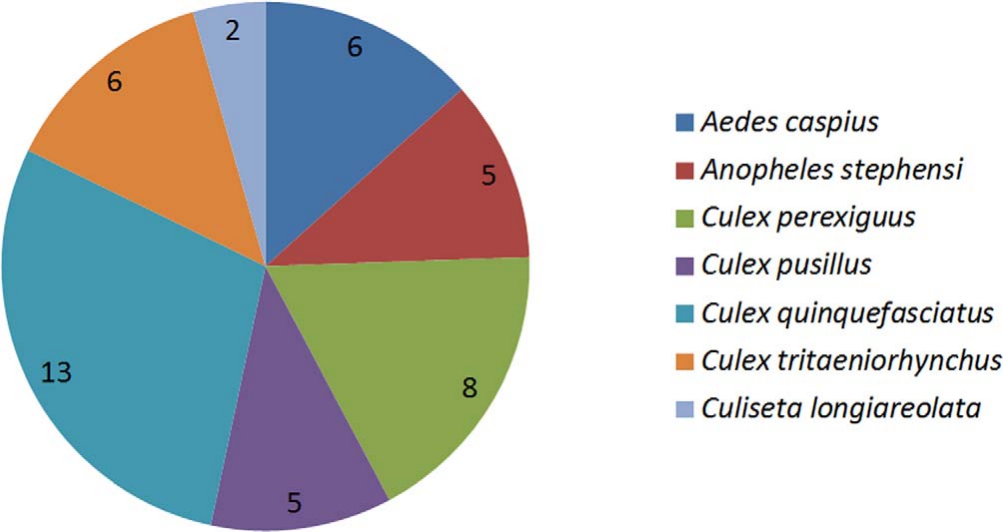
Numbers of positive sites for every mosquito species observed during our field surveys in Qatar, September 2017 and January 2019, by any sampling method, for a total number of 20 sites.

**Figure 4:**
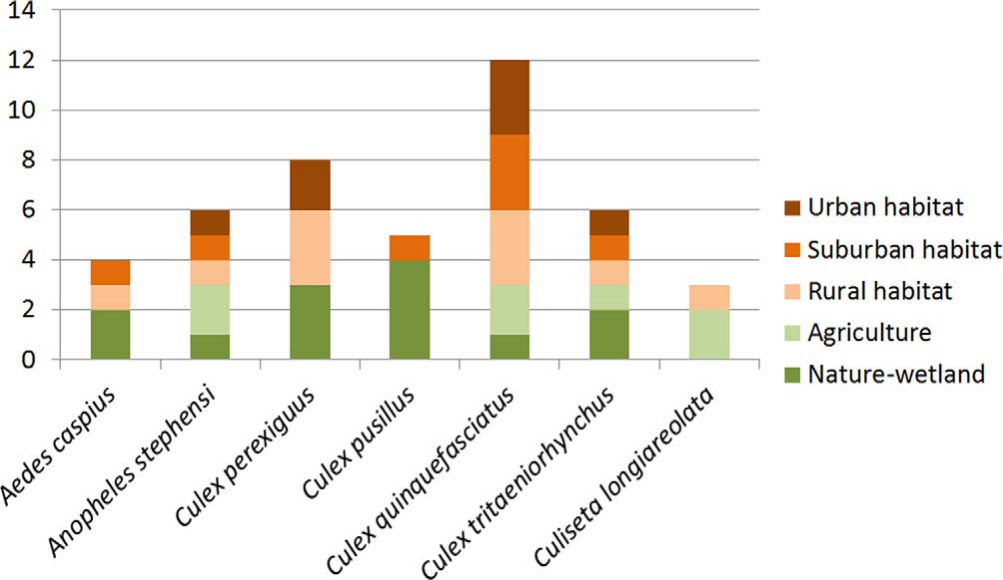
Numbers of positive sites for every land use category per mosquito species observed during our field surveys in Qatar, September 2017 and January 2019, by any sampling method, for a total number of 20 sites.

**Figure 5:**
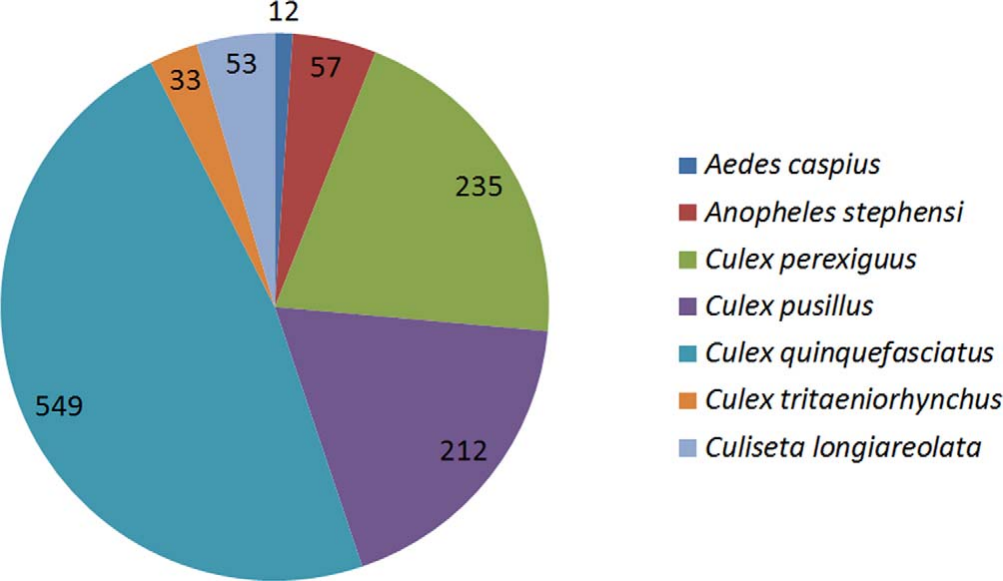
Relative proportions of mosquito species individuals collected during our field surveys in Qatar, September 2017 and January 2019, by any sampling method, for a total number of 1,151 individuals.

Seven mosquito species from four genera were observed: one *Aedes*, one *Anopheles*, four *Culex*, and one *Culiseta* (Table 5). All seven species were observed at both larval and adult (trapped or reared from immatures) stages, allowing accurate morphological identification. One specimen of *An. stephensi* (sample Q14b, adult female), two of *Cx. perexiguus* (samples Q04, adult male, and Q20, larvae) and one of *Cx. (Cux.) tritaeniorhynchus* Giles, 1901 (sample Q10, adult male) were submitted to molecular identification and obtained *COI* or *ITS2* sequences were compared with vouchers deposited in GenBank. Our *An. stephensi* sequence showed 100% similarity with specimens from Iran and Iraq; *Cx. perexiguus* sequences showed 100% identity with specimens from the United Arab Emirates, while the *Cx. tritaeniorhynchus* sequence showed >99% similarity with specimens from India, all confirming our morphological identification. Specimens of *Cx. quinquefasciatus* were also submitted to molecular identification. A total of 45 specimens (adults and larvae, 1–6 specimens per sample, from all samples harbouring *Cx. quinquefasciatus* except Q04 and Q19) were submitted to a PCR targeting the *ACE2* locus and all obtained band traces on the gel showed characteristic *Cx. quinquefasciatus* bands (274 bp). Preliminary genomic analysis also suggested that there is no notable trace of hybridisation with *Cx. pipiens* in the analysed genomes (Yuki Haba, pers. comm.). *Culex quinquefasciatus* was clearly the more abundant of the species, collected at 13 sites among 20 in total (Fig. 3), distributed in all land use categories (Fig. 4), and representing 48% of the collected individuals in total (Fig. 5). The lesser encountered species, *Cs. longiareolata*, was only found at two sites while all five remaining species were collected from five to eight different sites (Fig. 3). In terms of numbers of individuals, *Cx. perexiguus* and *Cx. (Barraudius) pusillus* Macquart, 1850 represented 20% and 18%, respectively, while the four remaining species represented less than 5%. Human landing catches revealed the occurrence of *Ae. caspius* only, while adult trappings also caught *Cx. quinquefasciatus* (88% of the caught individuals), *Cx. tritaeniorhynchus* (10%) and *An. stephensi* (1%), besides *Ae. caspius* (1%) (Fig. 5). Comparing the species composition according to land use categories showed that all categories have significant mosquito diversity with at least four species among the seven found here. All species but *Cs. longiareolata* were found to occur in wetlands, and all but *Cx. pusillus* in rural habitats. Similarly to *Cx. quinquefasciatus*, *Cx. tritaeniorhynchus* and *An. stephensi* were found in all land use categories (Fig. 4).

**Table 5:** Mosquito taxa reported to occur in Qatar in the literature, with date of first report, our findings, and assessed occurrence status. Black dots = confirmed presence.

Taxon	First report	References for Qatar	Session 1 2017–2018	Session 2 2017	Session 3 2019	Occurrence status
*Aedes aegypti*	1999	[2]				Introduced?
*Aedes caspius*	2009	[14, 15, 18]	●	●	●	Native
*Aedes dorsalis*	2015	[15]				1 Presence to be confirmed
*Anopheles culicifacies s.l.*	1999	[2]				1 Presence to be confirmed
*Anopheles multicolor*	1992	[11, 15, 18]				Native
*Anopheles sergentii*	1992	[11]				1 Presence to be confirmed
*Anopheles stephensi*	1999	[2, 14, 15, 18, 21]	●		●	Native
*Culex bitaeniorhynchus*	2015	[21]				1 Presence to be confirmed
*Culex laticinctus*	2015	[14]				1 Presence to be confirmed
*Culex mimeticus*	2015	[21]				1 Presence to be confirmed
*Culex pipiens*^2^ complex	1985	[1, 14, 15, 18, 19]				Native; Identity of occurring complex members to be confirmed
*Culex perexiguus*	2015	[14]			●	Native
*Culex pusillus*	2009	[14, 18]	●	●	●	Native
*Culex quinquefasciatus*	1988	[2, 12, 14, 15, 19]	●^3^		●	Native; Member of *pipiens* complex
*Culex sitiens*	2015	[14]				1 Presence to be confirmed
*Culex tritaeniorhynchus*	2015	[14, 15]	●	●	●	Native
*Culex univittatus*	2009	[14, 18]				Native; Identity to be confirmed by sequencing
*Culex vagans*	2015	[21]				1 Presence to be confirmed
*Culiseta* sp*.*	1985	[1]				1 May refer to *Cs. longiareolata*
*Culiseta longiareolata*	This study	–	●		●	Native
*Coquillettidia* sp*.*	2015	[21]				1 Presence to be confirmed
Total numbers:	21		6	3	7	

1 Single record;

2 Mentioned as *pipiens* complex or form *molestus*;

3 As a group of three possible species, *Cx. perexiguus*, *Cx. pipiens* and *Cx. quinquefasciatus*.

## Discussion

Highly accurate and up-to-date data about the presence and distribution of various vector species are needed by public health authorities to assess the potential threat and devise effective counter strategies for VBDs. In the present study, three field survey sessions were conducted between 2017 and 2019 with the primary aim of collecting data on geographical, topographical, and seasonal distribution of various species of mosquitoes, in different regions of Qatar.

### Field data outcomes

The samples from our entomological survey were collected from various sites to account for different factors that may influence the breeding capabilities and distribution of mosquitoes, including farms, garden centres, industrial areas, sewage lakes and sewage treatment plants, urban building areas, wetlands, worker houses, and zoos. In our survey, one or more species of mosquitoes were found at every inspected location, with the southern house mosquito species *Cx. quinquefasciatus* showing the widest geographical distribution. This is not surprising as this species is well adapted to breed in a wide range of habitats, from artificial collection of water in man-made containers to natural water bodies [7, 12]. Our overall findings were in accordance with the known preferences of the species [7, 12]. For example, the immature samples of *Cx. tritaeniorhynchus* and *Cx. perexiguus* were collected more frequently from flooded land than artificial containers, while specimens of *Cx. pusillus* and *Ae. caspius* were frequently found in wetlands with brackish water. However, we were surprised to find *Cx. pusillus* in a metallic cistern filled with fresh water. The *Cs. longiareolata* samples, both adults and immatures, were collected from four different sites. This is the first time *Cs. longiareolata* specimens were detected in Qatar. Wetlands and rural habitats showed the highest mosquito fauna diversity (six species among seven) in comparison to other habitats such as agricultural land, suburban and urban habitats, which harboured at least four species. All these findings are of public health significance in terms of risk for nuisance or potential for pathogen transmission.

### Critical review of the species list

No invasive species were found during our surveys. Despite large scale inspection of many man-made containers located in both urban and suburban habitat, our surveys did not find even a single sample of *Aedes (Stegomyia) aegypti* (Linnaeus 1762), suggesting that this species is potentially uncommon in Qatar. The occurrence of the yellow fever mosquito, *Ae. aegypti*, was reported in Qatar in a single reference without providing any sampling details [2]. Nevertheless, the presence of *Ae. aegypti* in Qatar is hardly surprising, as it is reported to breed in several neighbouring countries. We need to be watchful about its possible import into Qatar by being vigilant at places of entry for goods (port, airport, road crossings). Similarly, an investigation for the possible introduction and presence of another invasive species, the Asian tiger mosquito *Ae. (Stg.) albopictus* (Skuse, 1894), which also inhabits artificial collection of water (e.g. containers) should be performed. Additionally, authorities need to be especially vigilant since this species is spreading worldwide and is even found in the Middle East (e.g. in Iran, Gulf of Oman coast; [9]). Intense international trade makes its introduction possible, and the local climate looks suitable for its establishment [10].

Two brackish-water wetland mosquitoes are reported to occur in Qatar. The first, *Ae. caspius*, looks to be widespread in the country based on our findings (Table 5). Previous studies have also reported the presence of these species in Qatar for long time. It is possible that the population of this particular species may increase following rainfall or artificial accumulation of water in sewage lakes, and subsequently disperse over several kilometres and bite the human population, causing nuisance. A second species, *Ae. (Och.) dorsalis* (Meigen, 1830), which has been reported only once before [15], shares many morphological characters with *Ae. caspius.* This particular species if known to have a northern Holarctic distribution; however, it has never been reported from any other country in the Middle East except Iraq and Turkey [2, 22]. In addition, *Ae. caspius* adults show morphological variabilities, which could cause its misidentification as *Ae. dorsalis* [7]. Therefore, the present study recommends that the presence of *Ae. dorsalis* should be further studied in Qatar with sample collections, morphological observations and molecular identification.

Four *Anopheles* species are reported to inhabit Qatar (Table 5). The most frequently reported species, *An. stephensi*, was also observed in our study. While the presence of *An. (Cel.) multicolor* Cambouliu, 1902 is suggested by two field studies [15, 18], the two other species *An. (Cel.) culicifacies s.l.* Giles, 1901 and *An. (Cel.) sergentii* (Theobald, 1907) are listed without any field observation data [2, 11] and therefore their presence has to be substantiated.

The mosquito species belonging to the genus *Culex* are the most widespread mosquitoes in Qatar. In the Middle East, the *Culex pipiens* complex comprises the two forms *pipiens* and *molestus*, and *Cx. quinquefasciatus* [13, 22]. However, distinguishing these species by morphology is a difficult task that requires meticulous specimen examination [7]. In our study, all specimens were identified as *Cx. quinquefasciatus*, including by molecular examination. Several articles on the Qatari fauna refer to the *Cx. pipiens* complex [1, 18, 19], while others mention both *Cx pipiens* form *molestus* and *Cx. quinquefasciatus* to occur [14, 15]. Therefore, further sampling and molecular examination is recommended to confirm the identity of the *Culex pipiens* complex members in Qatar.

*Culex (Cux.) univittatus* Theobald, 1901 and *Cx. perexiguus* are two other closely related species that exhibit very similar external morphology at all life stages [7]. Both species have been reported in the Arabian Peninsula [12] as well as in Qatar [14, 18]. In our study, we identified only *Cx. perexiguus*, confirmed by molecular identification. As for the *pipiens* complex, there is unclear morphological differentiation and thus further molecular examination is recommended for specimens attributed by morphology to *Cx. univittatus* [20]. The presence of *Cx. pusillus* and *Cx. tritaeniorhynchus* in Qatar was confirmed by our field studies, whereas five other *Culex* species reported in the literature were not found viz. *Cx. (Oculeomyia) bitaeniorhynchus* Giles, 1901, *Cx. (Cux.) laticinctus* Edwards, 1913, *Cx. (Cux.) mimeticus* Noè, 1899, *Cx. (Cux.) sitiens* Wiedemann, 1828, and *Cx. (Cux.) vagans* Wiedemann, 1828. All of them except *Cx. vagans* do occur in the Arabian Peninsula [2, 12, 22], but to date, there has been only a single record in the literature and thus the occurrence of these five species in Qatar remains to be confirmed.

Lastly, there is only one official record of detection of *Culiseta* sp. (under its synonym *Theobaldia*) [1] and for *Coquillettidia* sp. in Qatar [21]. The mention of *Culiseta* may refer to *Cs. longiareolata* that we report here for the first time, and the presence of *Coquillettidia* sp. has to be further investigated.

### Recommendations to further explore local mosquito fauna

Additional and extended field surveys should be performed at regular interval to provide the most comprehensive knowledge about the mosquito fauna in Qatar. The most comprehensive strategy would be to undertake a field survey at as many sites as possible throughout the country, covering all kinds of environments and applying various sampling and trapping methods, more intensely during the rainy season but also the rest of year.

While city parks may not provide relevant mosquito fauna data because of their regular treatment by insecticides, wildlife conservation centres and animal holdings are important to investigate. In addition, surveys should focus on points of entry (ports, airports) as well as labour camps and industrial zones for possible alien species introductions. There are chances of discovering previously undetected mosquito species in Qatar given the existence of many other species in neighbouring countries (e.g. 36 species in Saudi Arabia [2]). However, the most pressing priority must be to design field surveys to confirm the existence of the mosquito species reported to occur in Qatar only by a single study/sample (Table 5). A quick way of achieving this could be re-analysis of the already collected specimens preferentially by a third party (providing the samples are preserved by the institutes after completion of field surveys) [13, 14, 19]. Another way of achieving this would be to sample at the same locations as mentioned by authors in those studies, possibly at the same time of year.

Besides mapping the mosquito population in Qatar, entomological surveys should also aim to evaluate the risk of mosquito-borne pathogen transmission by collecting data on distribution, abundance, seasonality and biting behaviour of species. Such surveys may focus on (1) *Anopheles* species as potential vectors of malaria parasites, (2) *Ae. aegypti* and *Ae. albopictus* as potential vectors of chikungunya, dengue, and Zika viruses, (3) *Ae. caspius* as a potential vector of Rift Valley fever virus, and (4) *Cx. pipiens* complex, *Cx. perexiguus*, *Cx. tritaeniorhynchus*, and *Cx. univittatus* as potential vectors of West Nile virus. Finally, cross-sectional and longitudinal data collections are needed to support the building of mid- and long-term surveillance and control strategies.

### Summary outcome and prospects

Our field studies have immensely extended the length, breadth, and depth of Qatar’s existing mosquito fauna database. Our field surveys were neither able to confirm nor refute the existence of *Ae. aegypti* in Qatar; however, given the extensive geographical coverage and length of sample collection, we can confidently say that *Ae. aegypti* is neither widespread nor abundant in Qatar. This suggests that there is a minimal risk for local transmission of dengue, chikungunya or Zika viruses. The malaria vector *An. stephensi* is widespread and common, including in urbanised areas, suggesting a risk of local transmission of malaria parasites. The wetland mosquito *Ae. caspius* is likewise widespread and is probably responsible for biting nuisance at certain periods of the year, also representing a risk of Rift Valley fever virus transmission. Several potential vectors of West Nile virus are present in Qatar. The species *Cx. quinquefasciatus*, commonly known as the southern house mosquito, was present most abundantly and this species is mostly responsible for the indoor biting nuisance. Regular field studies are needed to further address the knowledge gaps in terms of distribution, breeding and biting preferences of different mosquito species currently present in Qatar to accurately assess the risk of mosquito-borne diseases [8, 27].

## Conflict of interest

The authors declare that they have no competing interests.
